# Development of Analytical Methods for Multiple Phycotoxins in Fish Plasma

**DOI:** 10.1021/acsomega.6c02812

**Published:** 2026-07-30

**Authors:** Olivia S. Hernandez, Marta P. Sanderson, Robert J. Latour, Juliette L. Smith

**Affiliations:** Virginia Institute of Marine Science, William & Mary, P.O. Box 1346, Gloucester Point 23062, Virginia, United States

## Abstract

Certain harmful algal blooms (HABs) produce phycotoxins, which can be toxic to animals and to humans through seafood consumption. Data are currently lacking regarding the accumulation of phycotoxins in fish species. Quick and reliable methods are needed to assess toxin load in various upper trophic level fish species, both for diagnostics in fish mortality and prevention of human poisoning events. In this study, a method was developed for the extraction and cleanup of phycotoxins from plasma of juvenile sandbar sharks (*Carcharhinus plumbeus*) using ultrahigh-performance liquid chromatography-tandem mass spectrometry (UHPLC-MS/MS). Three cleanup methods were evaluated by QuEChERS, a dispersive SPE (d-SPE) method, and two national laboratory standards, solid phase extraction (SPE) and Hexane wash. The percent recovery (%*R*) of each was calculated across 14 different phycotoxins, both hydrophilic and lipophilic. Using generalized linear models, QuEChERS d-SPE *%R* was found to be similar to the national laboratory standard cleanup methods (SPE and Hexane wash). QuEChERS d-SPE had the highest %*R* at 100.7 ± 13.5% along with the most time efficient and second most cost-effective cleanup method of those tested. QuEChERS d-SPE was then validated using plasma from two species of bony fishes: Atlantic striped bass (*Morone saxatilis*) and blue catfish (*Ictalurus furcatus*) and optimized for all phycotoxins. Averaged across all three species, QuEChERS d-SPE had a %*R* of 94.8 ± 14.4%. This method provides a nonlethal way of sample collection to set a baseline of phycotoxin exposure in fish and illustrates that differences in sample cleanup between species may be necessary, which can help guide future monitoring, management, and public health efforts.

## Graphical abstract

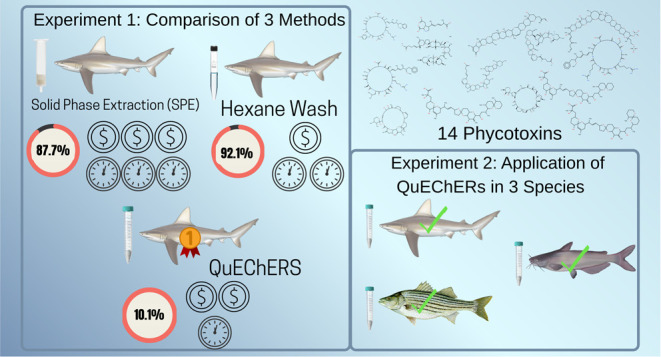

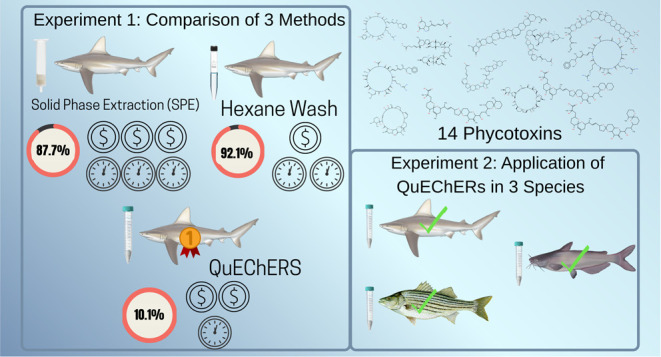

## 1 Introduction

The term harmful algal bloom (HAB) is generally defined as “micro- or macro- algae or cyanobacteria that cause adverse effects on biota and ecosystems”.^1^ These effects include adverse impacts on fish and wildlife, aquaculture, and fisheries, as well as tourism and human health. In the U.S., HABs are caused by a wide variety of algal species, including dinoflagellates and diatoms, and can occur in freshwater, saltwater, and brackish water. The frequency and geographic extent of HABs have increase in recent decades and this trend has been partially attributed to anthropogenic factors such as nutrient pollution, aquaculture expansion, and ocean warming. HAB impacts are expected to worsen under changing climate conditions.^1–3^

**Table 1:** Mean and Standard Deviation (SD) for % Recovery (%*R*) of All Phycotoxins in Sandbar Shark Plasma across Three Methods (SPE, Hexane, and D-SPE) Assessed in Experiment 1. AZA1 = Azaspiracid-1, AZA2 = Azaspiracid-2, AZA3 = Azaspiracid-3, DA = Domoic Acid, MC-LR = Microcystin-LR, MC-RR = Microcystin-RR, MC-YR = Microcystin-YR, DTX1 = Dinophysistoxin-1, DTX2 = Dinophysistoxin-2, GDA = Goniodomin-A, OA = Okadaic Acid, PbTx2 = Brevetoxin-2, PTX2 = Pectenotoxin-2, YTX = Yessotoxin

Method	Mean	SD
SPE	87.7	32.6
Hexane wash	92.1	12.2
d-SPE (QuEChERS)	100.7	13.5

Many HABs produce bioactive compounds, known as phycotoxins, that can affect human, aquatic animal, and environmental health. Phycotoxins have been documented in the Great Lakes, along both coasts of the U.S., the Gulf of Mexico, and globally.^4–10^ HABs can overlap in space and time, leading to coexposure of biota to multiple phycotoxins.^11–13^ In the U.S., the regulated marine phycotoxins include paralytic shellfish toxins (PSTs), amnesic shellfish toxins (ASTs), neurotoxic shellfish toxins (NSTs), diarrhetic shellfish toxins (DSTs), azaspiracid shellfish toxins (AZTs), and ciguatoxins. These phycotoxins are named based on the route of exposure to humans, often through consumption of contaminated shellfish^2,14^ or fish.^15,16^ Ingestion of marine phycotoxins can cause neurological, gastrointestinal, and hepatic symptoms in humans. Microcystins (MCs) are a class of phycotoxins prevalent in freshwater and brackish systems. Microcystins are a concern in the U.S. due to their hepatotoxicity and diarrhetic effects in humans, and as such, a tolerable daily intake for MC-LR has been published by the World Health Organization^17^ and health advisory levels have been set for drinking water by the U.S. Environmental Protection Agency.^18^ Several phycotoxins can also affect marine and freshwater fauna, including brevetoxins (PbTxs), pectenotoxins (PTXs), and goniodomin A (GDA).^19–21^ PbTxs are responsible for large, recurring fish kills along the west coast of Florida, where bloom-related mortalities are significant enough to alter community structure.^20,22^ Despite these impacts, however, data are lacking on the accumulation of most phycotoxins in fishes.

Elasmobranchs (sharks, skates, and rays) have been historically understudied in this context, even though their slow-growth, long lifespans, and low fecundity make them highly susceptible to population declines. Many species have been historically overfished and are currently listed as endangered or protected by the International Union for Conservation of Nature (IUCN).^23,24^ As mid- to upper-trophic level predators, these fishes have diverse diets and many undergo extensive seasonal migrations, potentially exposing them to a wide range of contaminants.^25^ Additionally, higher trophic level organisms can accumulate contaminants as they propagate up the food web. Sharks, in particular, have been shown to accumulate a multitude of contaminants, including persistent organic pollutants (POPs), polychlorinated biphenyls (PCBs), and heavy metals.^26–29^ To our knowledge, only four studies have assessed phycotoxin accumulation in sharks and rays, and all have been conducted in Florida, U.S.^13,30–32^ This limited research indicates that the field is relatively new and that substantial data gaps remain.

Current methods for sampling toxins in fishes are time-consuming, expensive, and frequently toxin specific. Typical approaches involve lethal sampling to collect organs such as the liver or brain. Muscle tissue, sampled as filet or by nonlethal muscle biopsy, has been tested as an alternative and can be useful for assessing toxin transfer potential to human consumers. However, quantifying toxins in muscle tissue may underestimate food web transfer potential, as muscle generally accumulates lower toxin concentrations than other organs (e.g., liver) due to the lipophilic nature of many phycotoxins.^33^ Plasma is another nonlethal matrix that can potentially be used for phycotoxin analysis. It provides a “snapshot” of recent environmental conditions experienced by an organism due to its faster turnover rate relative to muscle or liver.^34^ Sampling plasma also enables repeated, longitudinal monitoring of individuals. However, existing extraction methods for phycotoxin analysis of plasma were originally developed for human or rat samples,^35–37^ and method development for fish plasma have thus far focused on other contaminants.^38^

Development of a streamlined, cost-effective, and nonlethal method to extract toxins across a broad range of polarities from fish would greatly enhance monitoring and detection efforts in aquatic ecosystems. Current cleanup techniques used during phycotoxin extraction from complex biological matrices, such as solid phase extraction (SPE) and hexane washing, are time-consuming and expensive. A dispersive SPE (d-SPE) method originally developed for the analysis of pesticide residue in produce called Quick, Easy, Cheap, Effective, Rugged, and Safe (QuEChERS)^39^ has been used to analyze organochlorines, pharmaceuticals, and freshwater phycotoxins in both humans and fish.^40–46^ Recently, QuEChERS was successfully used during a study to analyze both freshwater and marine phycotoxins in juvenile bull sharks.^13^ Using d-SPE during phycotoxin extraction should increase the percent recovery while reducing costs and processing time.

The objective of this study was to develop a standard, nonlethal method of sample collection by extracting and cleaning up plasma samples from fish and analyzing for the presence of 14 hydrophilic and lipophilic phycotoxins by LC–MS/MS. The preferred method would be similar to or superior to the current standard methods while allowing for a more streamlined workflow. Three different cleanup methods were tested using percent recovery as a measure of efficacy, matrix effects were investigated, and the final method was optimized across three fish species, juvenile sandbar sharks (*Carcharhinus plumbeus*), and two species of bony fishes: Atlantic striped bass (*Morone saxatilis*) and blue catfish (*Ictalurus furcatus*).

## 2 Experimental Section

This study was conducted in two experiments. The first experiment was conducted to validate the use of plasma for phycotoxin analysis and to compare the extraction efficiency using d-SPE as the cleanup method against those of the two national laboratory standard methods. The second experiment was to expand the d-SPE method to plasma samples from two species of bony fishes: the recreationally important Atlantic striped bass and the invasive, bottom-dwelling blue catfish.

### 2.1 Reagents and Analytical Phycotoxin Standards

All organic solvents used for extraction, sample cleanup, and mobile phases were of LC–MS grade (Honeywell Burdick & Jackson, MI, USA). Ultrapure water was prepared using a Barnstead Genpure system (Thermo Fisher Scientific, Waltham, MA, USA). Mobile phase additives were either Optima LC/MS grade (Thermo Fisher Scientific) or eluent additives for LC–MS (Honeywell Fluka, Charlotte, NC, USA). The following biotoxin certified reference materials were purchased from the National Research Council Canada: pectenotoxin-2 (PTX2), okadaic acid (OA), dinophysistoxin-1 (DTX1), dinophysistoxin-2 (-DTX2), yessotoxin (YTX), azaspiracid-1 (AZA1), azaspiracid-2 (AZA2), azaspiracid-3 (AZA3), and domoic acid (DA). Standards for brevetoxin-2 (PbTx2) and microcystins –RR, -YR, -LR (MC-RR, -YR, LR) were purchased from Abcam (ab143469) and Sigma-Aldrich (33578-1 ML), respectively. Goniodomin A (GDA) was purified from cultures of *Alexandrium monilatum*.^47^ QuEChERS d-SPE cleanup kits were manufactured by ThermoFisher Scientific (kit #S2-2-GFV-AOAC-KIT).

### 2.2 Plasma Sample Collection

To sample fish across a range of habitats, diets, life history traits, and physiology, plasma was collected from three fish species: neonate (<70 cm) sandbar sharks, Atlantic striped bass, and blue catfish. Together the three species span different habitats, life history traits, and diets, meaning their blood differs physiologically,^48–50^ leading to possible matrix effects and varying recovery efficiencies. All samples were collected during routine surveys conducted from June—August 2023 by the Virginia Institute of Marine Science (VIMS). Survey collections were conducted in Virginia, U.S. at randomly selected sites in the major tributaries of Chesapeake Bay (Atlantic striped bass, blue catfish; protocol # 2022-0041) and in the bay mainstem and coastal lagoons of the Eastern Shore (sandbar shark; protocol # 2023-0099). Blood was drawn from the caudal vein of live animals using an 18-G, 11/2-in. hypodermic needle and 5 ml syringe. Approximately 1 mL of blood per kg of body weight was collected in a 6 mL plastic blood collection tube (lithium heparin 95 USP units), stored on ice in a dark cooler during transport to the lab, and centrifuged at 1570*g* for 6 min at room temperature to separate the plasma. The plasma was then decanted into a 15 mL centrifuge tube and stored in a −20 °C freezer until extraction.

### 2.3 Experiment 1: Comparison of Three Cleanup Methods in Shark Plasma

Because of the complex nature of plasma as a matrix, cleanup is required between the initial extraction and analysis by UHPLC-MS/MS. Three methods of cleanup were tested: traditional SPE, hexane wash, and d-SPE using QuEChERS media. Each cleanup method was performed in triplicate using pooled plasma homogenates from at least six individual sandbar sharks to minimize individual variability.

#### 2.3.1 Plasma Extraction Method

All plasma samples were suspended in 90% aqueous methanol (MeOH) at a 1:4 sample/solvent ratio (equivalent to 1 mL of plasma and 4 mL of 90% MeOH) and sonicated for 30 min at room temperature in the dark using a bath sonicator. Following sonication, one of the following three cleanup procedures was performed on the raw plasma extract (Figure 1).

**Figure 1:**
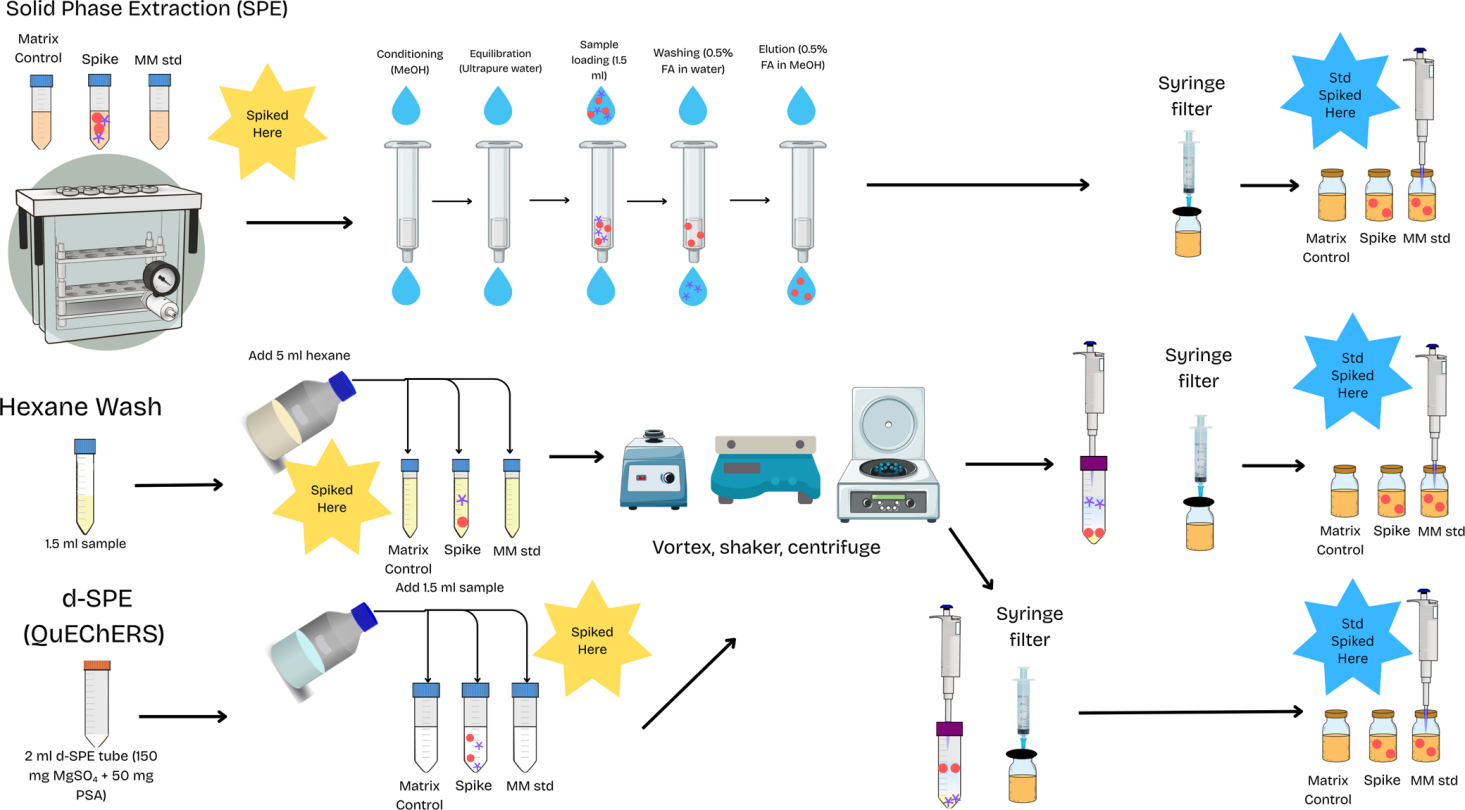
Design schematic showing the workflow of each cleanup method (SPE, Hexane wash, and d-SPE). Stars indicate at which step the extracts were spiked with certified reference material (CRMs) to track percent recovery (%*R*).

#### 2.3.2 Cleanup Method 1—SPE

Raw plasma extract was transferred into individual 50 mL centrifuge tubes in 1.5 mL aliquots and designated as either a matrix control (no phycotoxins added) or a matrix spike with a suite of 14 phycotoxins added before the SPE clean up. To assess plasma matrix effects, the same phycotoxins were prepared without a matrix, into 100% methanol (MeOH spikes) at the same final concentration as the matrix spikes. Final concentrations were 25 nanograms per milliliter (ng/mL) for PTX2, OA, DTX1, DTX2, YTX, DA, PbTx2, MC-RR, -YR, LR, and GDA, and 1 ng/mL for AZA1, AZA2, and AZA3. Controls received the same amount of MeOH without phycotoxins. For cleanup, SPE cartridges (60 mg, 3-cc Waters Oasis Hydrophilic–Lipophilic Balanced (HLB) SPE cartridges) were attached to a vacuum manifold and preconditioned with MeOH, followed by Ultrapure water. The HLB sorbent was chosen due to its ability to capture a wide range of hydrophilic and lipophilic compounds in the same method. All samples, including matrix controls and spikes, were diluted to 5% MeOH with acidified water (0.5% formic acid in Ultrapure water) to ensure DA would bind to the HLB sorbent during sample loading. Diluted, acidified extracts were loaded, and the cartridges were washed with acidified water before blowing the cartridges dry under a vacuum. Analytes were eluted from the SPE cartridge using two 0.75 mL aliquots of acidified MeOH (0.5% formic acid in MeOH). All samples were filtered using a 3 mL plastic syringe equipped with a 0.22 μm PVDF syringe tip filter (13 mm, Durapore). Following clean up and filtering, matrix controls were split, and a 0.9 mL aliquot was added to a separate vial to which the 14 phycotoxins were added, and these became the matrix-matched standards. All extracts were frozen at −20 °C until analysis.

#### 2.3.3 Cleanup Method 2—Hexane Wash

Raw plasma extract was transferred into separate 10 mL glass centrifuge tubes in 1.5 mL aliquots and designated as matrix controls, spikes, or matrix-matched standards. The matrix spikes and MeOH spikes were prepared as described in Cleanup [Sec sec2.3.2]. Each glass tube of sample extract had 5 mL of hexane added, and samples were vortexed for 1 min at high speed (setting 8–9) using a Scientific Industries Vortex-Genie 2. Samples were then centrifuged at 2000*g* for 10 min at 4 °C. The upper hexane layer was removed and discarded using disposable glass Pasteur pipettes. The remaining lower layer, consisting of the spike or matrix control extracts, was syringe filtered (0.22 μm PVDF, 13 mm, Durapore). Preparation of matrix-matched standards and freezer storage of extracts followed Cleanup [Sec sec2.3.2].

#### 2.3.4 Cleanup Method 3—d-SPE

Raw plasma extract was transferred into separate 2 mL d-SPE tubes (1.5-ml aliquots) prefilled with QuEChERS sorbent (150 mg MgSO_4_+50 mg PSA). Samples were designated as matrix controls, spikes, or matrix-matched standards. The matrix spikes and MeOH spikes were prepared as described in Cleanup [Sec sec2.3.2]. Samples were vortexed for 30 s at high speed (setting 8–9), followed by shaking for 15 min on a VWR Standard Shaker, Model 3500 (setting 6–7). Finally, samples were centrifuged at 3250*g* for 5 min at 4 °C to create a pellet with the dispersive SPE sorbent. The supernatant in the d-SPE tubes, consisting of matrix control or spike extracts, was syringe filtered (0.22 μm, PVDF, 13 mm, Durapore). Preparation of matrix-matched standards and freezer storage of extracts followed Cleanup Method 1.

### 2.4 Experiment 2: d-SPE Application in Multiple Species

To test the applicability of d-SPE beyond shark plasma, a second experiment was conducted with plasma from Atlantic striped bass and blue catfish.

d-SPE cleanup for each species was performed in triplicate using pooled plasma homogenate from at least six individuals to reduce any individual variability. All plasma samples were suspended in 90% aqueous methanol (MeOH) at a 1:4 sample/solvent ratio, equivalent to 1 mL of plasma and 4 mL of 90% MeOH. All samples were then placed in a Branson Bransonic M Mechanical Bath 5800, at room temperature, for 30 min in the dark. The raw plasma extract from the two bony fish species were extracted via the same d-SPE protocol as described in Cleanup [Sec sec2.3.4].

### 2.5 Phycotoxin Analysis

All extracts from Experiments 1 and 2, including matrix controls, spikes, matrix-matched standards, and MeOH spikes, were analyzed within 2 weeks of extraction and clean up. Analytical blanks were prepared using 100% MeOH. Matrix controls were analyzed to check for endogenous phycotoxins in the sandbar shark, Atlantic striped bass, and blue catfish plasma. Extracts and blanks were analyzed using ultrahigh-performance liquid chromatography-tandem mass spectrometry, with a trapping dimension and at-column dilution (UHPLC-MS/MS with trap/ACD). A Xevo TQD (Waters, Milford, MA, USA) equipped with electrospray ionization (ESI) was used with multiple reaction monitoring (MRM) for detection. The mass spectrometer was operated in ESI+ and ESI- modes. Chromatographic and mass spectrometer conditions followed those previously described.^51^ Each extract was analyzed for the peak area of 14 different phycotoxins: Azaspiracid toxins (AZTs): AZA-1, −2, −3, Diarrhetic shellfish toxins (DSTs): OA and DTX-1, −2, Freshwater toxins: MC-LR, –RR, -YR, and other toxins PbTx-2, DA, GDA, PTX-2, and YTX. Precursor > product transitions^12^ for quantification were used with the addition of DA: *m*/*z* 312.0 > 266.1, 30 V, 15 eV^51^ and AZA3: *m*/*z* 828.5 > 810.5, 30 V, 30 eV^52^. Injection volumes were all 50 μL. The inlet method included a 2 min elongated gradient hold^11^ and analytical blanks were run between extracts to confirm the absence of detectable carryover.

### 2.6 Percent Recovery and Matrix Effect Calculations

Percent recoveries for each of the 14 phycotoxins were calculated by comparing peak areas between matrix spikes (before clean up) and matrix-matched standards (MM std) for each clean up method

1 $$percent\;recovery\left(\%R\right)=\left(\frac{A_{spike}}{A_{MMstd}}\right)\times 100$$

where *A*_spike_ and *A*_MMstd_ are the peak areas in the matrix spikes and matrix-matched standards, respectively. Percent recovery (or analytical efficiency) represents the combined effects of extraction efficiency and matrix effects. Specifically, it reflects how well the phycotoxins (1) were extracted from plasma (sandbar shark—Experiment 1, Atlantic striped bass, and blue catfish plasma—Experiment 2) using each cleanup method and (2) detected within the matrix relative to the clean MeOH spike. Calculated percentages will be heavily influenced by signal enhancement or suppression arising from contaminants that were coextracted in each cleanup method.

Percent matrix effects were calculated for each of the 14 phycotoxins by comparing peak areas between matrix matrix-matched standards (after clean up) and MeOH spikes (no matrix) for each cleanup method

2 $$matrix\;effect\left(\%M\right)=\left(\left(\frac{A_{MMstd}}{A_{MeOHspike}}\right)-1\right)\times 100$$

where *A*_MM std_ is the peak area in the matrix-matched standard and *A*_MeOH spike_ is the peak area for MeOH spikes. Percent matrix effect quantifies how the matrix either suppresses or enhances the signal of the analyte, with negative values representing suppression and positive values representing enhancement relative to the MeOH spike (no matrix). Since matrix-matched standards are added after extraction and clean up, this calculation solely represents the analytical performance of the LC–MS/MS in a complex matrix relative to pure organic.

Matrix controls were analyzed to check for endogenous phycotoxins in each plasma matrix. If endogenous phycotoxins were detected in controls, their peak areas were divided by the peak areas of their corresponding spike samples which were then used to ensure that %*R* was not artificially enhanced.

### 2.7 Statistical Modeling

Generalized linear models (GLMs) were used to assess significant differences in percent recovery (%*R*, eq 1) among the three cleanup methods applied to the sandbar shark plasma (Experiment 1). GLMs are expressed as

3 $$g\left(\mu_{i}\right)=\beta_{0}+\sum_{j=1}^{p}\beta_{j}x_{ij},Var\left(y_{i}\right)=\phi V\left(\mu_{i}\right)$$

where μ_*i*_ = *E*(*y*_*i*_) is the expected value of the response for observation *i*, g(·) is the link function for the assumed probability distribution, *x*_*ij*_ are the predictor variables, β_*j*_ are the model coefficients, *V*(μ_*i*_) denotes the mean-variance relationship for the assumed distribution, and ϕ is the dispersion parameter.

Due to signal enhancement, several values of %*R* exceeded 100%. Consequently, the commonly used beta distribution modeling response variables defined on the (0,1) interval was not applicable. Therefore, several distributions within and outside the exponential family were evaluated for the response variable, including the Gaussian distribution (identity link function; classical ANOVA framework), the Student’s *t* distribution (log link; providing robustness to heavy-tailed data and outliers), and the log–normal, gamma, Tweedie, and inverse Gaussian distributions (log links; for modeling positive-valued responses with variance scaling with the mean). Model selection was based on AIC and BIC, as well as diagnostic checks of QQplots of residuals and plots of residuals against fitted values. Following selection of an appropriate distribution, three model parametrizations were fitted: the first included *phycotoxin*, *method*, and their interaction; the second included *phycotoxin* and *method*; and the third included only *method*. The same suite of information criteria and diagnostic assessments were then used to select the final model.

## 3 Results and Discussion

In this study, three cleanup methods for UHPLC-MS/MS analysis of shark plasma were compared for percent recovery and matrix effect. Results show that the use of the QuEChERS d-SPE cleanup method is comparable, if not better, than the two national standard methods for the detection of 14 different marine and freshwater phycotoxins spanning a wide range of molecular weights and polarities. d-SPE was subsequently validated in the extraction and cleanup of plasma from two species of bony fishes. Although the blood chemistries of the three fish species are broadly similar, they reflect distinct differences in certain biochemical parameters due to contrasting osmoregulatory strategies, metabolic rates, and habitat use. Because of its quick turnover rate, blood plasma is thought to represent recent contaminant exposure compared to tissues like the liver and muscle that are used to assess long-term exposure.^53,54^ Plasma is also sampled nonlethally, allowing for repeat sampling, and represents an ideal matrix for phycotoxin surveillance, particularly in endangered and protected species. d-SPE is versatile and applicable across regions and industries for monitoring and diagnostic purposes. Our results are the first to show that it performs comparably to established national laboratory standards (SPE and Hexane) but requires only a fraction of the cost and time. Faster, more comprehensive, and accessible detection methods can enhance responses to phycotoxin outbreaks in aquaculture and fisheries, ultimately supporting more-informed management decisions.

### 3.1 Experiment 1: Comparison of Three Cleanup Methods in Shark Plasma

All five candidate distributions adequately fit the %*R* data, as indicated by acceptable QQplots (Kolmogorov–Smirnov tests, *p* > 0.05) and the absence of discernible patterns in the mean-variance relationship. AIC and BIC comparisons favored the Tweedie distribution, which was subsequently used to test the fixed effects (Table S2).

All main effects were statistically significant (*p* < 0.05), indicating that the relative performance of the cleanup methods varied across the 14 phycotoxins. Model-predicted means showed that d-SPE had the highest estimated %*R* for seven phycotoxins (AZA1, AZA2, DTX2, GDA, PbTx2, PTX2, and YTX), the second highest for five phycotoxins (AZA3, DA, DTX1, MC-LR, and MC-YR), and the lowest for two phycotoxins (MC-RR and OA; Figure 2). Statistical significance, as inferred by nonoverlapping 95% confidence intervals, varied depending on the method-phycotoxin combination.

**Figure 2:**
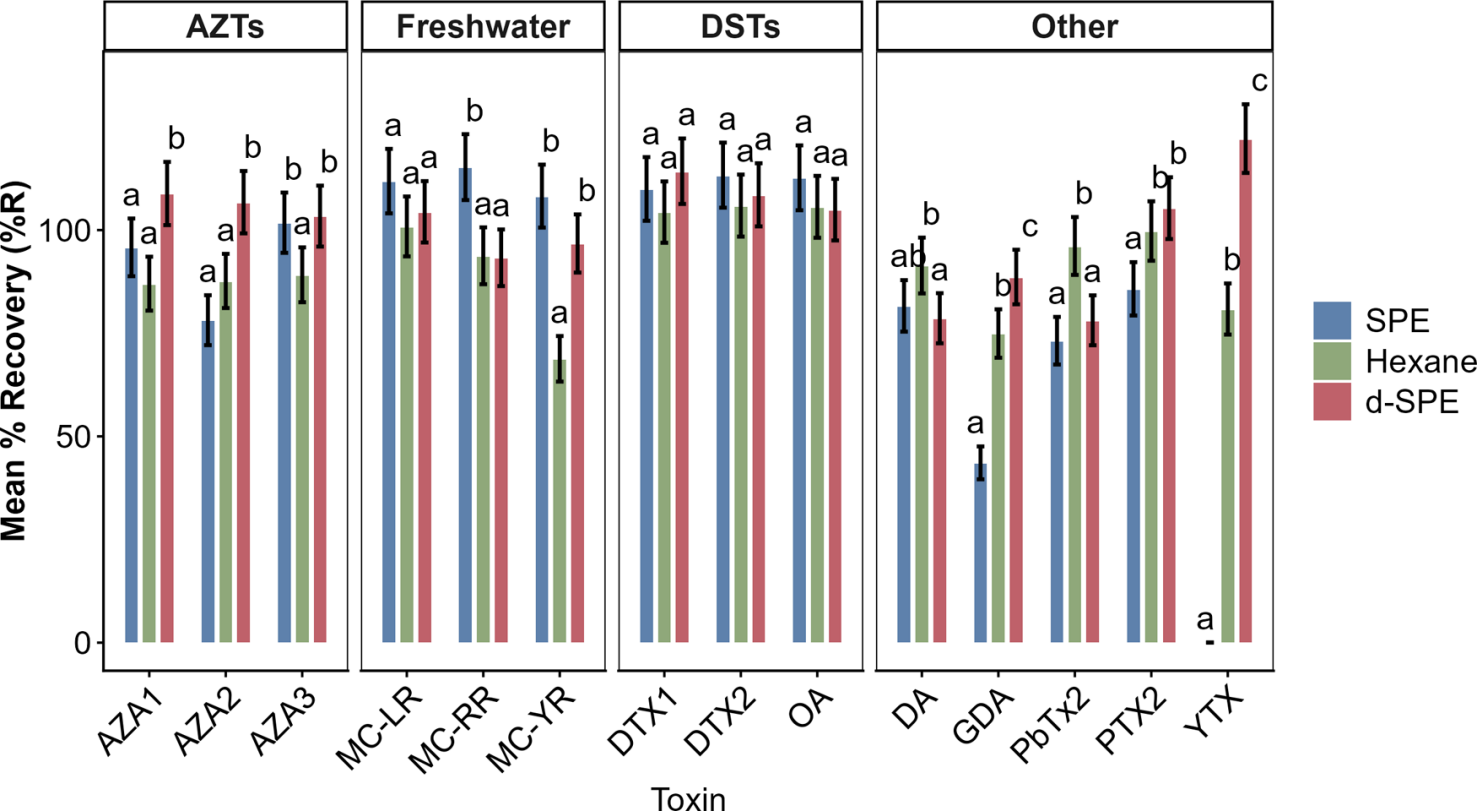
Predicted Mean % Recovery of phycotoxin in all three cleanup methods from neonate sandbar shark plasma samples (*n* = 3) spiked at 25 ng/mL (AZA’s at 1 ng/mL). Letters denote significant differences grouped by phycotoxin. Error bars are the 95% confidence interval, and the colors represent the three methods tested (SPE, Hexane , and d-SPE). AZA1 = azaspiracid-1, AZA2 = azaspiracid-2, AZA3 = azaspiracid-3, DA = domoic acid, MC-LR = microcystin-LR, MC-RR = microcystin-RR, MC-YR = microcystin-YR, DTX1 = dinophysistoxin-1, DTX2 = dinophysistoxin-2, GDA = goniodomin-A, OA = okadaic acid, PbTx2 = brevetoxin-2, PTX2 = pectenotoxin-2, YTX = yessotoxin.

Ignoring the significant interaction between cleanup method and phycotoxin (i.e., averaging recovery across all 14 phycotoxins), d-SPE had the highest overall mean recovery. Mean percent recoveries for SPE, Hexane, and d-SPE were 87.7 ± 32.6%, 92.1 ± 12.2%, and 100.7 ± 13.5%, respectively (Table 1). However, because cleanup method efficacy differed significantly among phycotoxins due to the method-phycotoxin interaction, these overall means should be interpreted with caution.

Because the cleanup method efficacy differed significantly among phycotoxins, pairwise comparisons were performed only among cleanup methods within each phycotoxin (Figure 2). Although significant differences among methods were detected for several phycotoxins, no consistent pattern of superiority was observed across the entire phycotoxin panel.

Therefore, d-SPE demonstrated an overall extraction efficacy in fish plasma that was similar to or better than the two national standard methods: Hexane and SPE. Beyond extraction efficacy, d-SPE was our preferred methods for logistical and economic reasons. First, it is much more time efficient than the SPE or Hexane wash method. For a batch of 9 plasma samples d-SPE took approximately 2–3 h to process whereas SPE and Hexane wash took approximately 6–8 h and 3–4 h/batch, respectively. It is more cost-effective than SPE, coming in at around $6.30/sample (not including CRM’s) while SPE costs around $9/sample. Despite Hexane wash being the most cost-effective at approximately $4/sample, the reduced accuracy (likely due to a precipitous protein layer during extraction), use of hazardous hexane, and time and effort ultimately leaves this method unsatisfactory for all 14 analytes. Finally, d-SPE is a simpler analytical method compared to SPE. During SPE, the analyte of interest binds to the SPE column while contaminants wash off, then is selectively eluted from the column, which leaves many steps for analyte to be lost. During d-SPE extraction, the dispersive SPE media simply binds to the contaminants in the sample and leaves the analyte in solution, creating far less opportunity for analyte loss.

The statistical significance of the *phycotoxin x method* interaction indicates that the choice of the cleanup step in sample purification should be guided by the specific analyte of interest. For example, %*R* of YTX was very low using SPE but higher in d-SPE and Hexane wash. This difference may be due to the acidification of the DI water used to dilute the samples to get DA to sorb to the HLB cartridges. This reduction in recovery from SPE may be due to the acidification of the DI water used to load the catrridge. YTX had a better recovery from HLB SPE under neutral load conditions (Garber, VIMS unpublished data). If both DA and YTX were target analytes, d-SPE or Hexane wash would be the preferred cleanup methods. Some analytes may require an additional cleanup step when present in a complex matrix, such as plasma. In this case, GDA had the lowest %*R* of all the phycotoxin tested across all three methods, while PbTx2 had extremely high levels of suppression (more than 75%). Both compounds are large, lipophilic molecules that tend to bind more strongly to blood proteins than hydrophilic compounds,^55^ which could contribute to their apparent loss. Formation of degradation products is also possible, although this remains unknown. Compounds similar to GDA and PbTx2 may need to include a protein precipitation step or use a spin filter to remove proteins prior to solvent extraction. If the analytes of interest were only large, lipophilic phycotoxins, then the solvent could be changed from MeOH to something less polar such as acetonitrile. Despite these sources of variability, d-SPE effectively captures a wide range of phycotoxins across different sizes and polarities and could be a useful tool in screening samples, particularly in areas where phycotoxin presence is unknown.

Endogenous DA was found in the shark plasma samples that were extracted using a Hexane wash. By dividing the peak area of the matrix control over the peak area of the matrix-matched standard, the endogenous DA was estimated to be 6–7% of the total recovery; that amount was subtracted from the percent recovery calculation for the Hexane method to avoid artificial inflation of recovery. Since DA was only found in the Hexane wash samples and had the highest DA recovery in Hexane wash, it does suggest that this method is the best for extracting and cleaning up samples when looking for DA. However, there were no limits of detection calculated for this study; therefore, further research is warranted. The presence of endogenous DA does, however, indicate that the neonate sandbar shark plasma collected contained low levels of DA in July 2024. It is important to note that the plasma was pooled from several sharks before extraction, and so, the concentration could have been higher within any one individual. Although it has been shown that juvenile leopard sharks (*Trakis semifasciata*) are resistant to neurotoxic effects from DA,^56^ further research is needed to determine whether resistance extends to other shark species, such as the sandbar shark, and other life stages.

### 3.2 Experiment 2: d-SPE Application in Multiple Species

The d-SPE method was applied to three species of fish to validate its efficacy in species spanning a wide range of life histories. The sandbar shark is a cartilaginous fish, a cosmopolitan species, and a diet generalist, the Atlantic striped bass is an anadromous teleost that is important both commercially and recreationally, and finally, the blue catfish is an invasive teleost that is largely recreational, and all are found in the Chesapeake Bay.^25,48,57–60^ While there were species- and phycotoxin-specific differences, the d-SPE method was able to successfully clean up the fish plasma matrix from all three species and allow for the detection of 14 different marine and freshwater phycotoxins.

The analysis of Atlantic striped bass and blue catfish plasma using d-SPE clean up revealed species-specific differences in phycotoxin-matrix interactions. Both species showed substantial amount of variability in %*R* for individual phycotoxin when using the same method. Across all three species the mean percent recovery for all toxins was 94.8 ± 14.4% (Table S2). Matrix effects were calculated for the three fishes and Atlantic striped bass plasma consistently showed high levels of suppression across the phycotoxins tested, while sandbar shark plasma consistently exhibited low levels of suppression. PbTx2 showed elevated levels of suppression in the plasma of all three fish species (>75%) The mean percent matrix effect across all species was −14.6 ± 32.4% (Figure 3 and Table S2).

**Figure 3:**
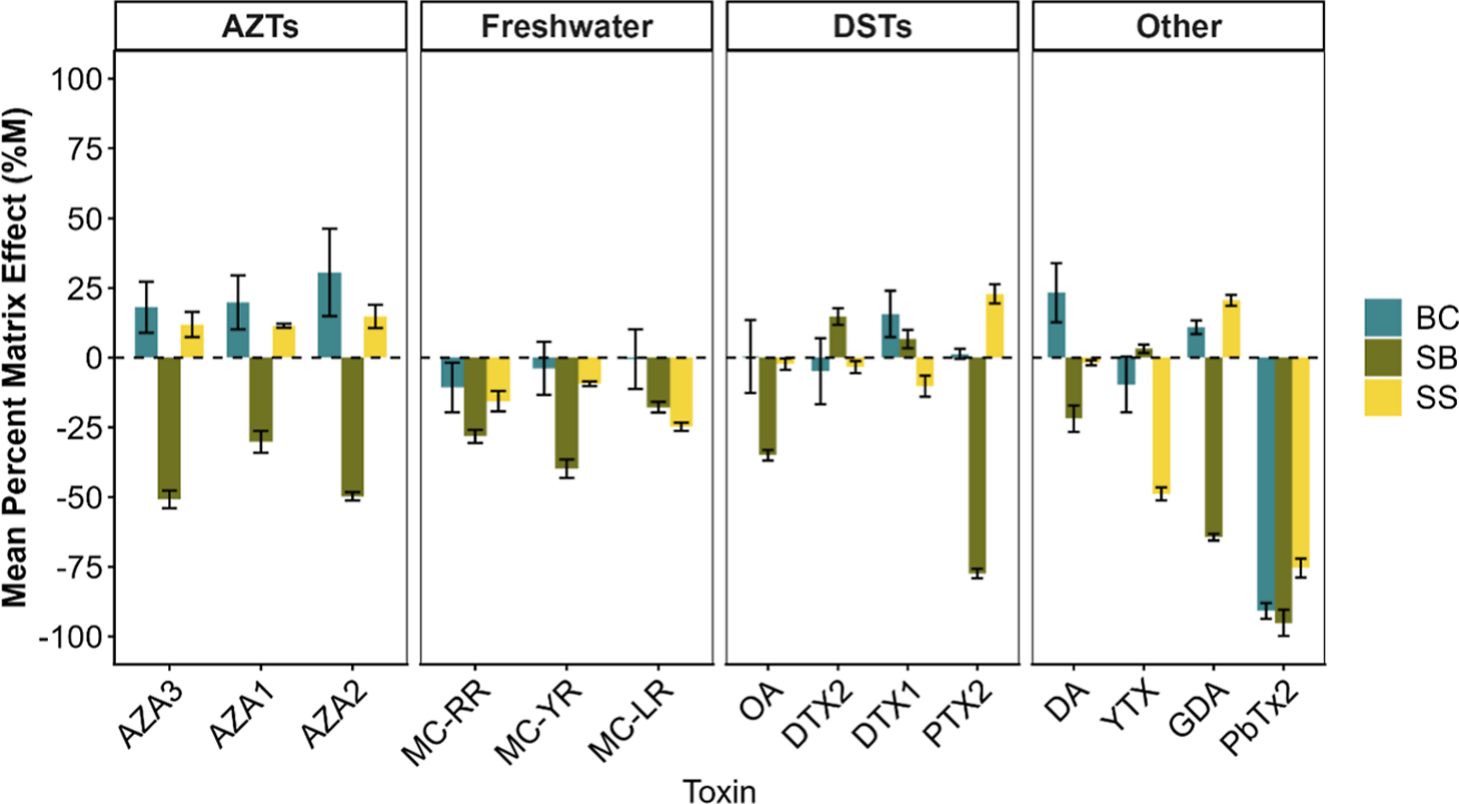
Mean percent matrix effect ±SD (%*ME*) in blue catfish (BC), Atlantic striped bass (SB), and sandbar shark (SS) plasma samples (*n* = 3) across 14 phycotoxins using the d-SPE method spiked at 25 ng/mL (AZA’s at 1 ng/mL). Positive values represent signal enhancement, and negative values represent signal suppression. AZA1 = azaspiracid-1, AZA2 = azaspiracid-2, AZA3 = azaspiracid-3, DA = domoic acid, MC-LR = microcystin-LR, MC-RR = microcystin-RR, MC-YR = microcystin-YR, DTX1 = dinophysistoxin-1, DTX2 = dinophysistoxin-2, GDA = goniodomin-A, OA = okadaic acid, PbTx2 = brevetoxin-2, PTX2 = pectenotoxin-2, YTX = yessotoxin.

The causes of these species-specific differences remain unclear but may be due to differences in the lipid or protein content in the blood. For these species, additional cleanup steps such as protein precipitation or spin filtering may help mitigate those interferences, but more research is needed for further validation. Data on the comparative physiology of teleost plasma are lacking, highlighting the need for more research. Studies often group individual species into larger taxonomic categories such as fish, multiple species of shellfish, or bivalves, but this study highlights the importance of understanding the physiology of study species, as interspecific variability can represent a significant source of uncertainty.

## 4 Conclusions

Harmful algal blooms can produce phycotoxins that pose risks to ecosystems and human health. Rapid, affordable, and reliable methods are needed to assess the presence of phycotoxins in fish and other marine organisms. This study developed a method for detecting 14 different marine and freshwater phycotoxins using nonlethal sample collection methods and streamlined cleanup steps for LC–MS/MS. The d-SPE approach performed comparably to the current national laboratory standards (SPE and Hexane wash), while requiring substantially less time and cost. This study also demonstrated that plasma can be a useful matrix for monitoring fish health, particularly protected species because collection is nonlethal. It can be used to help detect threats before they become detrimental to the environment.

This study also highlights the importance of taking into consideration analyte characteristics, species physiology, and methodological differences in cleanup procedures prior to sample collection.

In the future, the analytes of interest should be considered when designing the methodology to determine whether protein precipitation, spin filtering, or acetonitrile elution may be necessary. Testing d-SPE across matrices beyond plasma, such as muscle tissue, liver tissue, or whole water samples, would help determine its broader applicability. Moreover, investigating interspecies differences in matrix effects and blood chemistry of species of interest can help inform decision-making when determining how to process samples.

## Supplementary Material



## Notes

Recovery and matrix effect data for all three fish species and AIC and BIC values for all 6 model distributions tested (PDF)
