# Supplementary material for: Development of Analytical Methods for Multiple Phycotoxins in Fish Plasma
Source: ACS Omega. 2026 Jul 30;11(31):46172–80. doi: 10.1021/acsomega.6c02812 (PMC13470688; doi:10.1021/acsomega.6c02812)
Supplement: Supplementary file 1 [file ao6c02812_si_001.pdf]

Development of Analytical Methods for Multiple Phycotoxins in Fish Plasma

Olivia S. Hernandez, Marta P. Sanderson, Robert J. Latour, Juliette L. Smith*

Virginia Institute of Marine Science, William & Mary, P.O. Box 1346, Gloucester Point, VA, USA 23062

Supporting Information

Table S1. ΔAIC, ΔBIC and degrees of freedom (df) values for models. M1 = Gaussian, M2 = Student's *t*, M3 = lognormal, M4 = Gamma, M5 = inverse Gaussian, M6 = Tweedie.

Model	dAIC	dBIC	df
M6	0	0	44
M2	16.9	14.1	43
M1	30.5	27.7	43
M3	52.6	49.8	43
M4	160.4	157.6	43
M5	1202.1	1199.3	43

Table S2. Mean percent recovery ± standard deviation (%R±SD) of each phycotoxin from the plasma of each species (sandbar shark, blue catfish, and Atlantic striped bass), and mean percent matrix effect ± standard deviation (%ME±SD).

Toxin	Species	%R± SD	%M ± SD
**DA**	Sandbar	78.3±8.8	-1.9±0.9
	Blue Catfish	87.8±22.6	23.3±10.6
	Striped Bass	77.7±10.1	-22.0±4.7
**MC-RR**	Sandbar	93.0±8.0	-15.7±3.7
	Blue Catfish	98.1±3.6	-10.8±8.9
	Striped Bass	88.9±5.1	-28.3±2.4
**MC-YR**	Sandbar	96.5±1.5	-9.4±0.8
	Blue Catfish	92.2±14.5	-3.9±9.5
	Striped Bass	92.6±2.3	-39.9±3.3
**MC-LR**	Sandbar	104.2±1.6	-24.8±1.5
	Blue Catfish	97.3±10.8	-0.5±10.7
	Striped Bass	100.3±5.9	-17.8±1.9
**AZA3**	Sandbar	103.1±6.1	11.9±4.5
	Blue Catfish	99.7±7.9	18.1±9.2
	Striped Bass	97.9±17.9	-50.9±3.2
**AZA1**	Sandbar	108.6±1.2	11.5±0.7
	Blue Catfish	97.3±10.8	19.8±9.7
	Striped Bass	96.1±3.7	-30.3±4.1
**AZA2**	Sandbar	106.4±4.7	14.8±4.1
	Blue Catfish	99.1±13.5	30.6±15.7
	Striped Bass	84.9±8.1	-49.8±1.5
**OA**	Sandbar	104.7±6.8	-2.4±2.1
	Blue Catfish	95.2±21.1	0.4±13.1
	Striped Bass	79.9±7.1	-35.1±1.9
**DTX2**	Sandbar	108.3±7.4	-3.4±2.2
	Blue Catfish	92.5±10.1	-4.9±11.9
	Striped Bass	106.0±3.0	14.7±3.0

**YTX**	Sandbar	121.8±8.34	-48.9±2.3
	Blue Catfish	96.0±30.5	-9.6±10.0
	Striped Bass	90.3±2.6	3.2±1.5
**DTX1**	Sandbar	114.0±6.4	-10.3±3.8
	Blue Catfish	104.4±25.0	15.7±8.3
	Striped Bass	101.4±6.0	6.7±3.3
**PTX2**	Sandbar	105.1±3.9	22.9±3.5
	Blue Catfish	82.8±17.3	-13.1±1.8
	Striped Bass	84.8±11.2	-77.4±1.7
**GDA**	Sandbar	88.4±2.6	20.6±2.0
	Blue Catfish	68.7±10.8	10.9±2.4
	Striped Bass	37.7±1.9	-64.4±1.2
**PbTx2**	Sandbar	77.9±13.0	-75.4±3.4
	Blue Catfish	120.5±64.4	-90.8±2.9
	Striped Bass	80.3±99.4	-95.1±4.7
